# Development of a reverse-genetics system for murine norovirus 3: long-term persistence occurs in the caecum and colon

**DOI:** 10.1099/vir.0.042176-0

**Published:** 2012-07

**Authors:** Armando Arias, Dalan Bailey, Yasmin Chaudhry, Ian Goodfellow

**Affiliations:** Section of Virology, Department of Medicine, Imperial College London, Norfolk Place, London, W2 1PG, UK

## Abstract

Human noroviruses (HuNoV) are a major cause of viral gastroenteritis worldwide, yet, due to the inability to propagate HuNoV in cell culture, murine norovirus (MNV) is typically used as a surrogate to study norovirus biology. MNV-3 represents an attractive strain to study norovirus infections *in vivo* because it establishes persistence in wild-type mice, yet causes symptoms resembling gastroenteritis in immune-compromised STAT1^−/−^ mice. The lack of reverse-genetics approaches to recover genetically defined MNV-3 has limited further studies on the identification of viral sequences that contribute to persistence. Here we report the establishment of a combined DNA-based reverse-genetics and mouse-model system to study persistent MNV-3 infections in wild-type (C57BL/6) mice. Viral RNA and infectious virus were detected in faeces for at least 56 days after inoculation. Strikingly, the highest concentrations of viral RNA during persistence were detected in the caecum and colon, suggesting that viral persistence is maintained in these tissues. Possible adaptive changes arising during persistence *in vivo* appeared to accumulate in the minor capsid protein (VP2) and the viral polymerase (NS7), in contrast with adaptive mutations selected during cell-culture passages in RAW264.7 cells that appeared in the major capsid protein (VP1) and non-structural protein NS4. This system provides an attractive model that can be readily used to identify viral sequences that contribute to persistence in an immunocompetent host and to more acute infection in an immunocompromised host, providing new insights into the biology of norovirus infections.

## Introduction

Gastroenteritis remains one of the top five causes of death worldwide and the second most common cause in low-income countries (Monroe, 2011). The development of a rotavirus vaccine places human noroviruses (HuNoV) as the leading cause of food-borne disease and non-bacterial gastroenteritis worldwide (Kahan *et al.*, 2011; Koo *et al.*, 2010). It has been estimated that HuNoV infections in developing countries result in >200 000 deaths and >900 000 hospitalizations of children younger than 5 years every year (Patel *et al.*, 2008). In high-income countries, however, low mortality rates are reported for HuNoV, with the major impact being observed on elderly and immunocompromised patients. It is estimated that 23 million symptomatic infections occur each year in the USA alone, resulting in at least 300 deaths (Harris *et al.*, 2008; Mead *et al.*, 1999; van Asten *et al.*, 2011). Frequent large outbreaks caused by HuNoV are reported during the winter season, affecting the operational capacity of many closed environments (e.g. schools, hospitals, military camps). Large economic losses (estimated to be >£100 million in the UK alone) are therefore associated with hospital disruptions caused by HuNoV outbreaks (Lopman *et al.*, 2004). In addition to acute gastroenteritis, noroviruses have recently been linked to a number of significant clinical diseases, such as the exacerbation of inflammatory bowel disease (IBD) (e.g. Crohn’s disease, ulcerative colitis), seizures in infants and other neurological disorders (Byrnes & Griffin, 2000; CDC, 2002; Chen *et al.*, 2009; Ito *et al.*, 2006; Khan *et al.*, 2009). Recent studies carried out with animal models have supported the connection between norovirus infection and the exacerbation of IBD (Cadwell *et al.*, 2010; Lencioni *et al.*, 2008).

The lack of efficient systems to cultivate HuNoV in cell culture has limited studies on norovirus replication and pathogenesis. Discovery of the closely related murine norovirus (MNV), capable of replication in several cell lines, has provided an ideal alternative with which to study the molecular biology of noroviruses and the identification of antiviral strategies to control them (Karst *et al.*, 2003; Wobus *et al.*, 2004). MNV-1 was the first strain to be isolated and was found to lead to a rapid systemic and lethal infection in immunocompromised STAT1^−/−^ mice (Karst *et al.*, 2003). MNV-1 replicates in both immunocompromised and wild-type mice, although in the presence of a competent immune system, virus replication is rapidly controlled and cleared, becoming undetectable in faeces and organs from 7 days post-infection (Hsu *et al.*, 2006; Kahan *et al.*, 2011; Karst *et al.*, 2003; Mumphrey *et al.*, 2007). Following the identification of MNV-1, three new MNV strains were isolated from asymptomatic mice from different geographical location colonies, referred to as MNV-2, -3 and -4. These three new strains were able to establish persistent infections in wild-type ICR (imprinting control region) mice that lasted for >10 weeks and resulted in large amounts of viral RNA being shed in the faeces (Hsu *et al.*, 2006). Subsequent studies confirmed that MNV is highly prevalent in research colonies and that the different strains isolated are genetically closely related and form a single serotype (Barron *et al.*, 2011; Hsu *et al.*, 2007; Thackray *et al.*, 2007).

**Fig. 1.**
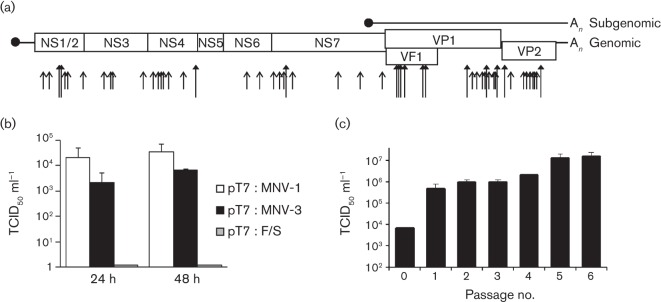
Recovery of MNV-3 by reverse genetics. (a) Schematic representation of the MNV-3 genomic and subgenomic RNAs. MNV-3 contains four ORFs. Translation of ORF1 results in the synthesis of a viral polyprotein that is then processed proteolytically into different mature non-structural viral proteins as indicated. Translation of the subgenomic RNA results in the synthesis of major capsid protein VP1 (ORF2), minor capsid protein VP2 (ORF3) and virulence factor VF1 (ORF4). The sequence of pT7 : MNV-3 differs from the previously reported sequence in 53 nt positions along the genome, represented as short arrows with non-filled arrowheads (synonymous) and as long arrows with filled arrowheads (non-synonymous). (b) TCID_50_ virus titres obtained after recovery of pT7 : MNV-1, pT7 : MNV-3 and pT7 : F/S (pT7 : MNV with a frameshift mutation) in BSR-T7 cells 24 and 48 h post-transfection, as explained in the text. (c) Virus titres of MNV-3 samples resulting from serial passage of MNV-3 recovered in RAW264.7 cells. MNV-3 recovered in (b) was used to infect RAW264.7 cells at a low m.o.i. (0.001). MNV-3 produced (passage 1) was used for serial passage at a low m.o.i. of 0.1 (passages 2–5) or 0.3 (passage 6) in RAW264.7 cells.

The MNV genome is a positive-stranded RNA molecule around 7.5 kb in length, containing four ORFs (Fig. 1a). Viral RNA replication, catalysed by the viral RNA-dependent RNA polymerase NS7, results in the generation of new viral genomes, as well as a subgenomic RNA encompassing the ORF2–ORF4 coding region. Replication is primed by VPg (NS5), which also plays a key role in recruiting host factors to initiate VPg-dependent viral protein synthesis (Chaudhry *et al.*, 2006; Daughenbaugh *et al.*, 2003, 2006; Goodfellow *et al.*, 2005). Translation of ORF-1 leads to the synthesis of a large polyprotein that is cleaved into the different non-structural mature proteins (NS1–7) (Sosnovtsev *et al.*, 2006). Translation of the subgenomic RNA leads to production of the capsid proteins VP1 and VP2 (ORF2 and 3, respectively), as well as the innate immune antagonist VF1 (ORF4) (McFadden *et al.*, 2011; Wobus *et al.*, 2006).

The development of several reverse-genetics approaches for MNV has allowed molecular characterization of the viral and host factors that regulate norovirus replication (Chaudhry *et al.*, 2007; Ward *et al.*, 2007; Yunus *et al.*, 2010). We have shown previously that genetically defined wild-type MNV-1 and mutant viruses recovered in cell culture can establish an acute infection in mice, which has been instrumental for the molecular characterization of MNV-1 in cell culture and *in vivo* (Bailey *et al.*, 2008, 2010; McFadden *et al.*, 2011). The recent identification of other MNV strains able to establish stable persistent infections in mice has opened new avenues to understanding viral sequences that may contribute to viral persistence. However, the absence of reverse-genetics approaches for these new MNV variants has limited the establishment of an animal model to study persistent infections in mice with a genetically defined MNV isolate.

Here, we describe for the first time the combination of reverse-genetics approaches and animal models to study the establishment of a persistent norovirus infection in wild-type C57BL/6 mice. Viral RNA was detected in faeces for at least 56 days. In addition, all infected animals displayed high MNV-3 RNA loads in the caecum and colon, suggesting that these are the major sites for persistent replication.

## Results

### Reverse-genetics recovery of MNV-3

To enable the generation of a full-length cDNA clone of MNV-3, a sample of MNV-3 provided by Robert Livingston (Hsu *et al.*, 2007) was used to infect RAW264.7 cells and total RNA was extracted 24 h post-infection as explained in Methods. This collected sample represented the viral population after three rounds of infection at low m.o.i. in RAW264.7 cells. The full viral genome was then amplified by RT-PCR using primers that introduced a truncated T7 RNA polymerase promoter at the 5′ end and a tail of 26 adenines at the 3′ end. Cloning of the amplified fragment into the backbone plasmid used previously to generate an MNV-1 reverse-genetics system (Chaudhry *et al.*, 2007) resulted in the insertion of a self-cleaving δ-ribozyme sequence after the A_26_ sequence to ensure the generation of a defined 3′ end (Walker *et al.*, 2003). A number of constructs were sequenced in their entirety and screened for their ability to produce infectious virus (data not shown). Surprisingly, the consensus sequence and the sequences of three individual constructs displayed >50 common sequence alterations with respect to the initially reported MNV-3 sequence (Hsu *et al.*, 2007).

We selected pT7 : MNV-3 clone 2 as our reference clone, referred to here as pT7 : MNV-3 (GenBank accession no. JQ658375), which contained 53 sequence differences compared with the MNV-3 sequence of Hsu *et al.* (2007) (Fig. 1a). These changes are the likely result of additional passage of the virus in cell culture. The pT7 : MNV-3 construct was used to recover infectious MNV-3 in cell culture as described previously (Chaudhry *et al.*, 2007). Briefly, BSR-T7 cells previously infected with a recombinant fowlpox virus expressing recombinant T7 polymerase (FPV-T7) were transfected with pT7 : MNV-3 or a similar wild-type MNV-1-containing construct (pT7 : MNV-1). Virus titres obtained at both 24 and 48 h post-transfection were noticeably lower for the MNV-3 clone (2.1×10^3^ and 6.3×10^3^ TCID_50_ ml^−1^) than for MNV-1 (2.1×10^4^ and 3.5×10^4^ TCID_50_ ml^−1^) (Fig. 1b), but recovery was reproducible and could also be obtained using *in vitro*-transcribed and enzymically capped RNA as described previously (Yunus *et al.*, 2010) (data not shown).

### MNV-3 derived entirely from cDNA can be propagated successively in RAW264.7 cells

MNV-3 recovered above, referred to as passage 0 (MNV-3 p0), was used to infect RAW264.7 cells at an approximate m.o.i. of 0.001 TCID_50_ per cell. Ninety-six hours post-infection, cytopathic effect was not apparent. Infectious MNV-3 passage 1 (MNV-3 p1) was released by two successive freeze–thaw cycles and the filtered lysate was used to inoculate a new monolayer of RAW264.7 cells (m.o.i. 0.1). MNV-3 p2 was recovered at 48 h post-infection, associated with large degrees of cytopathic effect. Consecutive passages of MNV-3 at m.o.i.s ranging from 0.1 to 0.3 resulted in an increase in the virus titre and in the cytopathology in RAW264.7 cells during infection. Virus yields (titres) by passage 6 (MNV-3 p6) were typically 15-fold higher than at passage 2 (Fig. 1c). This was also followed by an increase in the appearance of cytopathic effect (not shown).

To determine whether increased virus yield was associated with changes in the viral genome, viral RNA was extracted from MNV-3 p2 and p6 stocks and their full genomes were amplified by RT-PCR. Sequence analysis revealed only two sequence changes in MNV-3 p6 relative to the cDNA clone and in both cases a degree of heterogeneity was observed; typical chromatogram signal intensities were in the range 60–80 %/40–20 % for the mutant/wild-type sequence. The mutations identified were A2269G and C5957U, resulting in the changes D50G and T301I in the viral NS4 and VP1 proteins, respectively. No mutations were found in the MNV-3 p2 genome, suggesting that changes in MNV-3 p6 may reflect tissue-culture adaptation. We and others have previously observed that, during repeated passage of MNV-1 in RAW264.7 cells, similar adaptation occurs, with changes also being observed in NS4 and VP1 (V11I and K296E, respectively; Bailey *et al.*, 2008; Wobus *et al.*, 2004).

### MNV-3 recovered by reverse genetics establishes persistent infections in C57BL/6 mice

**Fig. 2.**
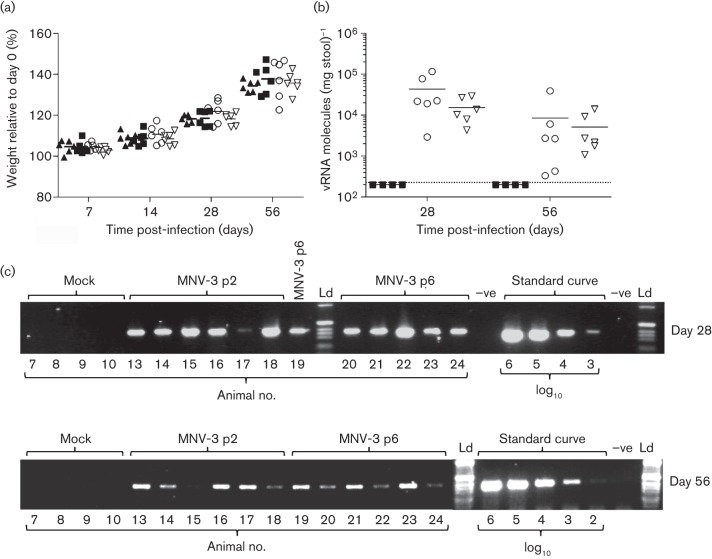
MNV-3 recovered by reverse genetics establishes persistent infections in mice. C57BL/6 mice, 4–5 weeks of age, were inoculated with 100 µl sample containing 1.0×10^5^ TCID_50_ MNV-3 p2 or 1.5×10^6^ TCID_50_ MNV-3 p6. Controls included uninfected and mock-infected animals. (a) No significant differences were observed in the weight gain of inoculated animals (○, p2; ▽, p6) compared with controls (▴, uninfected; ▪, mock-infected). (b, c) Viral RNA was extracted from stool pellets collected (○, p2; ▽, p6; ▪, mock-infected) and was detected by quantitative (b) and semiquantitative (c) RT-PCR in all infected animals, but not in mock-infected animals. Quantitative and semiquantitative PCRs were performed as described in the text. Horizontal bars in (a) and (b) represent mean values. −ve, Negative control; Ld, DNA ladder.

To investigate whether MNV-3 recovered in cell culture from cDNA was able to establish a persistent infection in immunocompetent mice, as shown previously for field isolates of MNV-3 in ICR mice (Hsu *et al.*, 2006), we performed oral inoculations of 4–5-week-old C57BL/6 male mice. The maximum possible dose of virus was used in this initial trial, namely 100 µl of either MNV-3 p2 or MNV-3 p6 (1.0×10^5^ and 1.5×10^6^ TCID_50_, respectively). C57BL/6 mice were chosen due to the ready availability of a wide number of knockout and knock-in lines in this genetic background. Inoculated animals showed no clinical symptoms or differences in weight increase relative to mock-infected or uninfected animals for the whole period of surveillance (56 days) (Fig. 2).

To determine whether the cDNA-derived virus had established a persistent infection, stool samples were collected from the animals at days 28 and 56 post-infection, and analysed by both quantitative and semiquantitative RT-PCR. Two different methodologies were used for further validation of the levels of viral RNA and to overcome limitations observed with the detection limits of each assay. We detected genomic MNV RNA in the faeces of all the inoculated animals at both days 28 and 56 post-infection by semiquantitative RT-PCR, but not in faeces of mock-infected animals (Fig. 2). Semiquantitative and quantitative analyses were in agreement with each other, and showed that most animals excreted viral RNA at day 28 in the range of 10^5^ molecules (mg stool)^−1^ [corresponding to approx. 10^4^ molecules of viral RNA (µl sample analysed)^−1^] (Fig. 2). At day 56, a decrease in viral RNA levels was observed when compared with the same animals at day 28 [approx. 10^3^–10^4^ molecules (mg stool)^−1^; *P*<0.05, two-way ANOVA], suggesting that virus replication had been partially restricted. Although not statistically significant, mean values for animals infected with MNV-3 p2 tended to be higher than in animals infected with MNV-3 p6. Sequence analysis of MNV-3 VP1 in faeces of three different animals infected with MNV-3 p6 (day 56) indicated that the dominant sequence present at amino acid position 301 in VP1 was T. This result might suggest that adaptive mutation T301I, selected during passage in RAW264.7 cells and present in the passage 6 stock but absent after two passages, resulted in decreased fitness *in vivo* and was outcompeted by wild-type variants, detected as a minority in MNV-3 p6.

### MNV-3 infection occurs at a low inoculating dose and shows fast replication kinetics in mice

**Fig. 3.**
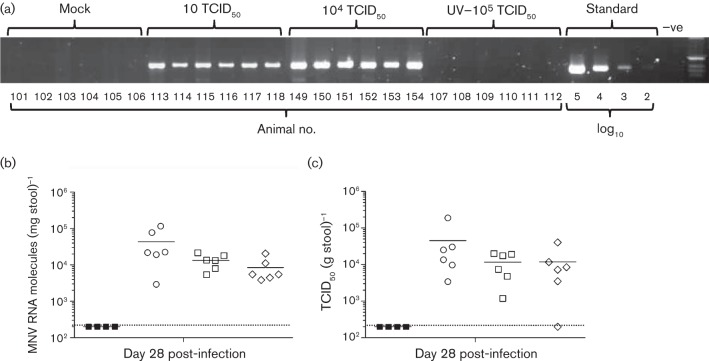
Low-dose inoculation of MNV-3 results in establishment of viral persistence. C57BL/6 mice 4–5 weeks of age were inoculated with 100 µl sample containing 10^4^, 10^3^, 10^2^ or 10 TCID_50_ MNV-3, 10^5^ TCID_50_ UV-inactivated MNV-3, or filtered cell-culture lysate (Mock). (a) Animals infected with 10 or 10^4^ TCID_50_ MNV-3 showed detectable viral RNA in faeces by day 28 post-infection. Animals infected with 10^2^ or 10^3^ TCID_50_ were also positive (not included in the figure). Animals mock-inoculated with UV-inactivated MNV-3 or cell-culture lysate presented no detectable viral RNA. (b) Viral RNA levels in faeces collected at 28 days post-infection from animals mock-infected (▪) or infected with 10 (◊), 10^4^ (□) or 10^5^ (○) TCID_50_ MNV-3 (from Fig. 2). (c) Titration by TCID_50_ in RAW264.7 cells of infectious virus isolated from faecal samples of animals shown in (b). Horizontal bars in (b) and (c) represent mean values.

To investigate the infectivity of MNV-3 in an immunocompetent background *in vivo*, we inoculated C57BL/6 mice with 10-fold-increasing doses of MNV-3 p2, ranging from 10 to 10^4^ TCID_50_ (Fig. 3). At 28 days post-infection, the four groups of animals inoculated with virus displayed detectable viral RNA by RT-PCR, whereas mock-infected animals or animals inoculated with 10^5^ TCID_50_ UV-inactivated MNV-3 were negative for MNV RNA (Fig. 3 and data not shown). Hence, the ID_50_ (amount required to infect 50 % of the animals) of MNV-3 is likely to be <10 TCID_50_. We carried out the quantification of viral RNA in animals previously infected with 10^4^ and 10 TCID_50_ by quantitative PCR, and compared them with the values obtained previously for animals inoculated with 10^5^ TCID_50_ (Figs 2 and 3). The three groups of animals displayed similar mean values, although animals infected with 10^5^ TCID_50_ presented higher concentrations on average (although not significant by statistical analysis, one-way ANOVA test).

To determine whether the viral RNA detected in the faeces correlated with the presence of infectious virus in the stool pellets, we carried out TCID_50_ assays for the samples analysed by quantitative PCR (Fig. 3c). We detected infectivity in the stool pellets of 17 out of 18 animals. In the positive samples, virus titres ranged from 1.2×10^3^ to 1.9×10^5^ TCID_50_ (g stool sample)^−1^. The calculated virus titres per stool pellet (20–50 mg on average) ranged from 5.2×10^1^ to 6.1×10^3^ TCID_50_ shed in every faecal sample after 28 days post-infection. Interestingly, all animals initially inoculated with 10 TCID_50_ MNV-3 p2 (with the exception of animal 118, which was negative for infectivity) released >200 TCID_50_ in a single pellet.

**Fig. 4.**
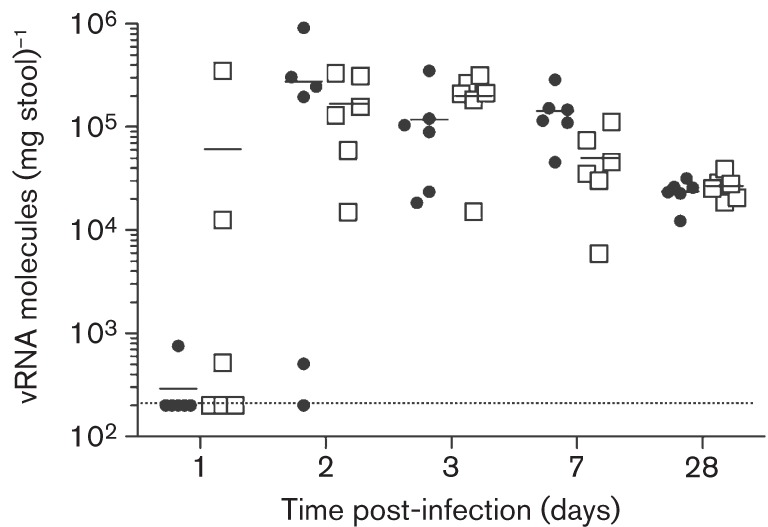
MNV-3 shows fast kinetics of replication in mice. Groups of six mice were infected with a low dose of MNV-3 [10 (•) or 10^2^ (□) TCID_50_] and secretion of virus was monitored at days 1, 2, 3, 7 and 28 post-infection. MNV-3 shows fast replication kinetics, reaching maximum levels of RNA molecules in stools by day 2 post-infection. Establishment of infection is dose-dependent, with three of six animals inoculated with 10^2^ TCID_50_, and only one of six animals inoculated with 10 TCID_50_, being positive for virus shedding at 1 day post-inoculation. Horizontal bars represent mean values.

Given the low inoculating dose required to establish an MNV-3 infection *in vivo*, we decided to investigate the replication kinetics of MNV-3 after low-dose inoculation. Mice were inoculated with either 10 or 10^2^ TCID_50_ and the kinetics of viral RNA shedding in the faeces of each animal infected were determined by quantitative PCR. The data indicated that MNV-3 infection occurs rapidly, with all animals actively secreting viral RNA on day 3 (Fig. 4).

### MNV-3 persists in the caecum and large intestine

**Fig. 5.**
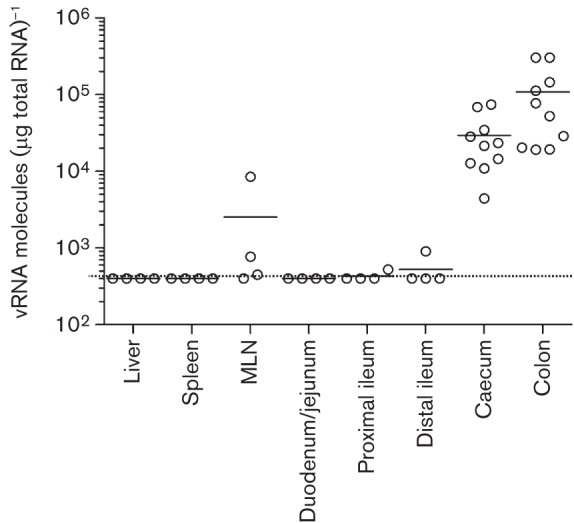
MNV-3 persists in the caecum and the colon. High levels of MNV RNA were detected in caecum and colon samples of animals 28 days post-inoculation. Lower amounts of viral RNA were detected in MLN and small intestine of some animals. MNV RNA levels in liver and spleen were below the detection limit [<4×10^2^ viral RNA molecules (µg total RNA)^−1^]. The organs and tissues collected included liver, spleen, MLN, different 1 inch sections of the small intestine comprising dudodenum/jejunum (1–2 inches from the stomach end), proximal ileum (3–4 inches from the stomach end) and distal ileum (the last inch before the caecum), caecum and colon. Horizontal bars represent mean values; the dotted line represents the limit of detection.

To identify the major tissue(s) for MNV-3 replication and persistence in C57BL/6 mice *in vivo*, we extracted various organs previously identified to support MNV replication. RNA was then isolated from the spleen, liver, mesenteric lymph node (MLN), different sections of the small intestine (duodenum/jejunum, proximal and distal ileum), caecum and colon from infected animals at 28 days post-inoculation. Strikingly, the highest levels of viral RNA were detected in the colon followed by the caecum, with mean values >10^5^ and >10^4^ MNV molecules (µg total RNA)^−1^, respectively (Fig. 5). Semiquantitative RT-PCR was also used to confirm these observations (not shown). MNV RNA was also detected in the MLN of some animals, but at considerably lower levels than in tissues from the large intestine.

### Persistent replication of MNV-3 in C57BL/6 mice results in the selection of repeated mutations in NS7 and VP2

**Fig. 6.**
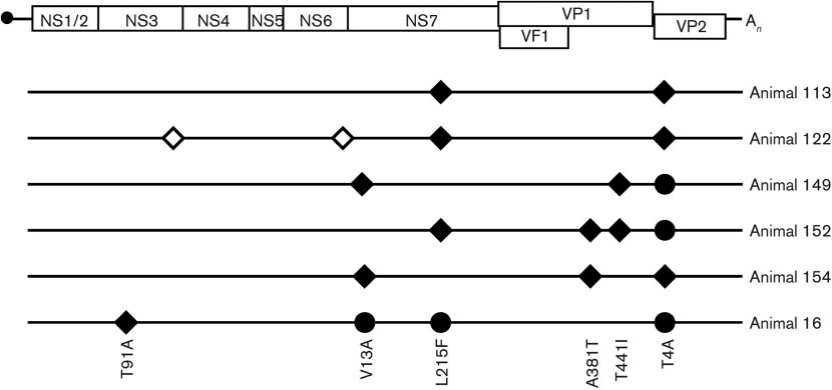
Sequence analysis of viral RNA from stools at days 28 (animals 113, 122, 149, 152, 154) and 56 (animal 16, which presented the highest viral RNA concentration at that time point). Empty and filled symbols represent synonymous and non-synonymous substitutions, respectively. ◊/⧫ represent mutations not totally imposed in the consensus sequence (30–80 % relative to wild-type sequence based on chromatogram quantification); • represents a totally imposed change.

To determine whether persistent replication of MNV-3 in mice resulted in the selection of adaptive mutations in the viral genome, we sequenced genomic RNA extracted from the faeces of five different animals at day 28 post-infection and from one animal at day 56 post-infection (Fig. 6). We identified three mutations repeated in three or more different animals: U3574C, C4179U and A6690G, which resulted in amino acid changes V13A and L215F in the NS7 polymerase and T4A in the VP2 minor capsid protein. In addition, only three other non-synonymous mutations were identified in the analysis: A381T and T441I in VP1, found in two clones, and T91A in NS3, found in one clone (Fig. 6).

## Discussion

Here, we describe the establishment of a persistent infection *in vivo* with MNV-3 recovered from cDNA by reverse genetics. To our knowledge, this is the first time that a persistent infection of C57BL/6 mice with MNV recovered from cDNA has been achieved. However, during the course of this study, a reverse-genetics system for the CR6 strain of MNV, shown previously to establish a persistent infection (Thackray *et al.*, 2007), has been described. Recombinant virus derived from this system has to date only been used to identify virulence determinants in the VP1 protein within a STAT1^−/−^ model (Strong *et al.*, 2012).

The combination of an efficient reverse-genetics system for MNV-3 and an animal model to study stable persistent infections constitutes a significant step forward in the development of systems for understanding norovirus molecular biology and pathogenesis. Persistent infections in humans by HuNoV have also been reported, although they mainly affect immunocompromised individuals (Capizzi *et al.*, 2011; Ludwig *et al.*, 2008). Nevertheless, recent data indicate that asymptomatic norovirus prevalence is approximately 12 % in the UK, although whether this represents subclinical acute infections or long-term asymptomatic secretion of virus is not known (Phillips *et al.*, 2010). A recent report has shown that MNV-3 infection in STAT1^−/−^ mice results in an acute infection with typical signs of gastroenteritis (i.e. delayed gastric emptying, changes to diarrhoeal symptoms) (Kahan *et al.*, 2011). This, combined with the fact that MNV-3 can establish a persistent infection in C57BL/6 mice, the most commonly used strain for the generation of knockout mice, provides additional support for the use of MNV-3 for the study of norovirus biology.

The observation that high viral RNA levels were detected in caecum and colon at day 28 post-infection, but not in the small intestine or other organs (MLN, spleen, liver), represents one of the most significant findings of this study. These data suggest that MNV-3 establishes a persistent infection in the large intestine, allowing efficient virus shedding in faeces over a prolonged time frame. The strong association between ulcerative colitis, a colon disease, and the presence of HuNoV during exacerbated disease (Khan *et al.*, 2009) supports the idea that the colon may also play an important role for HuNoV persistence. Two additional studies have provided further evidence for the connection between various colon disorders and active secretion of HuNoV in faeces, specifically in children with persistent diarrhoea and in patients with chronic diarrhoea associated with leukaemia (Capizzi *et al.*, 2011; Vernacchio *et al.*, 2006). A recent study comparing MNV-1 and MNV-3 *in vivo* has shown that the viruses replicate in different organs, including the caecum and the colon, but they are rapidly cleared, being undetectable by day 7 post-infection (Kahan *et al.*, 2011). Possible explanations for these apparently conflicting data may be the sensitivity of the assays used and differences in the experimental set-up, i.e. age and strain of mice. It is also worth noting that, whilst the MNV-3 used here was derived from cDNA, that used in the previous study was a virus passed in tissue culture several times.

Interestingly, MNV persistent infections have also been established in 7- to 15-week-old mice using the CR6 strain, with viral RNA also being detected in colon 14 days post-infection at levels lower than those observed in the ileum and MLN (Cadwell *et al.*, 2010). The results presented in this study differ somewhat from these results, which may be a reflection of the differing virus strain under study (CR6 versus MNV-3) or the age of the animals used, i.e. 7–15 weeks versus 4–5 weeks in our study. Alternatively, MNV replication for longer periods (>14 days) may have resulted in clearance from the ileum and MLN, but not from the caecum and the colon.

Analysis of full MNV-3 genome sequences derived from serial passage of MNV-3 in RAW264.7 cells and persistent replication in mice resulted in marked differences in genome evolution. Serial passage of MNV-3 in cell culture selected for non-synonymous mutations in NS4 and VP1. We have previously reported the selection of mutations in NS4 and VP1 during MNV-1 cell-culture passage: K296E in VP1 and V11I in NS4. K296E in MNV-1 VP1 was shown to cause attenuation in STAT1^−/−^ mice (Bailey *et al.*, 2008; Strong *et al.*, 2012). Interestingly, MNV-3 and MNV CR6, both causative of persistent infections in wild-type mice, encode E296 in VP1 instead of K. The substitution E296K in MNV CR6 resulted in non-recoverable virus, which may indicate that this mutation is not tolerated in the context of the CR6 VP1 (Strong *et al.*, 2012). Position 296 lies in close proximity to position 301 in the structure of VP1, both at the tip of the protruding P2 domain (Taube *et al.*, 2010), which we interestingly observed to be mutated (T301I) during repeated passage of MNV-3 in RAW264.7 cells. Our observations would suggest that, upon inoculation of animals with a mixed population of viruses containing both T and I at position 301, namely MNV-3 p6, only T at position 301 in MNV-3 VP1 persists *in vivo*. Clearly, further studies are warranted to examine the role of this position in tissue-culture adaptation and the possible associated fitness cost *in vivo*. In addition, adaptive mutations in the picornavirus homologue of NS4 (3A) have been linked to increased virulence in cell culture and/or *in vivo* (Arias *et al.*, 2010; Harris & Racaniello, 2005; Núñez *et al.*, 2001), warranting further studies on the precise function of the norovirus NS4 protein.

Persistent replication of MNV-3 *in vivo* did not select for any of the substitutions found during cell-culture passage, but instead selected for changes in VP2 and NS7. The mutations T4A in VP2, and V13A and L215F in NS7 were found in six, four and three animals, respectively. Additional mutations identified include A381T and T441I in VP1, and T91A in NS3. The T4A substitution in VP2 was found in the faeces of all animals analysed, but not during the serial passage of MNV-3 in RAW264.7, suggesting that this change may be important *in vivo* but not relevant for cell-culture replication. For the related feline calicivirus, the VP2 protein is essential for virus replication and for the assembly of infectious particles (Sosnovtsev *et al.*, 2005). Recent studies with HuNoV VP1 and VP2 have revealed co-evolution of both proteins in a time-dependent manner, highlighting the integral role of the VP2 protein in the norovirus life cycle (Chan *et al.*, 2012). Strikingly, we have found that alanine at position 4 in VP2 is absolutely conserved in >30 natural MNV isolates analysed. Nevertheless, the presence of threonine at position 4 in VP2 had no effect on virus recovery by reverse genetics or on virus replication in cell culture, being stably maintained after multiple passages in RAW264.7 cells. This highlights that the VP2 sequence may contribute to virus fitness *in vivo* due to selective pressures not observed in cell culture.

Mutations V13A and L215F in the viral RNA polymerase NS7 (Fig. 6) appear to affect polymerase surface residues, distant from the catalytic site, in the palm and fingers domains, respectively (Lee *et al.*, 2011). Given their accessibility, these changes may be affecting the interaction of NS7 with potential viral or cellular factors, although this hypothesis requires further investigation. Interestingly, substitutions found in VP1 at lower frequency (A381T and T441I) are predicted to lie within exposed residues of flexible loops situated in the apical region of P2 and P1 domains, respectively, which could be indicative of selection by neutralizing antibody response. In particular, A381 is situated in loop E′-F′, identified previously as a major immunodominant epitope with antibody-escape mutants being mapped in position 386 (Lochridge & Hardy, 2007; Taube *et al.*, 2010).

In summary, we have described for the first time the establishment of a persistent infection of C57BL/6 mice with MNV-3 recovered by reverse genetics. MNV-3 derived from cDNA persistently replicated in C57BL/6 mice for at least 56 days and was associated with high viral loads in the caecum and the colon. Preliminary data indicate that viral RNA is shed by infected mice for >6 months, emphasizing the ability of recovered MNV-3 to establish long-term persistent infections *in vivo* (N. McFadden, A. Arias, I. Goodfellow & P. Simmonds, unpublished results). This model may open new possibilities to study norovirus infections *in vivo* and different disorders associated with norovirus persistence.

## Methods

### 

#### Ethics.

Studies with C57BL/6 mice were performed in the St Mary’s CBS Unit of Imperial College London (PCD 70/2727) after ethical review by the Imperial College Ethical Review Panel and subsequent approval of the UK Home Office (PPL70/6838). All animal procedures and care conformed strictly to the UK Home Office Guidelines under The Animals (Scientific Procedures) Act 1986.

#### One-step construction of an infectious cDNA clone of MNV-3.

To enable the generation of a full-length cDNA clone of MNV-3, a sample of MNV-3 provided by Robert Livingston (University of Missouri, Columbia, MO, USA) was used to infect RAW264.7 cells at an m.o.i. of 0.1 TCID_50_ per cell (Hsu *et al.*, 2007). RNA was extracted from the infected cells 24 h post-infection. This sample collected represented the viral sequence after three rounds of low-m.o.i. infection in RAW264.7 cells. RNA was copied into cDNA using SuperScript II (Invitrogen) and a reverse primer containing a 3′ *Spe*I site, a poly(A) of 27 nt and the last 36 nt of the MNV-3 genome. Genomic MNV cDNA was fully amplified using KOD polymerase (Merck), the primer used for reverse transcription and a 5′ primer containing a *Spe*I site, a truncated T7 RNA polymerase promoter and the first 35 nt of the MNV genome. Gel-purified PCR product was digested with *Spe*I and ligated into the pT7 : MNV-1 backbone vector digested with *Spe*I and *Nhe*I. The resulting pT7 : MNV-3 contains 53 sequence changes (GenBank accession no. JQ658375) with respect to the originally published MNV-3 sequence (Fig. 1) (Hsu *et al.*, 2007).

#### Cells, infections and reverse-genetics recovery of MNV-3.

Baby hamster kidney cells expressing T7 RNA polymerase (BSR-T7) obtained from Klaus Conzelmann (Ludwig-Maximilians-Universitat München, Germany) (Buchholz *et al.*, 1999) were cultured in Dulbecco’s modified Eagle medium (DMEM) with 10 % FCS, 100 U penicillin ml^−1^ and 100 µg streptomycin µl^−1^. RAW264.7 cells, grown in DMEM with 10 % FCS, 100 U penicillin ml^−1^, 100 µg streptomycin µl^−1^ and 10 mM HEPES pH 7.6, were used for the titration (TCID_50_) and propagation of MNV-3.

For the recovery of infectious MNV-3, BSR-T7 cells were infected with a helper fowlpox virus expressing recombinant T7 RNA polymerase (FPV-T7) and transfected with pT7 : MNV-3 as described previously for MNV-1 (Chaudhry *et al.*, 2007). Generated MNV-3 infectious particles were released from BSR-T7 cells by two consecutive freeze–thaw cycles and supernatants were filtered. For the amplification and generation of MNV-3 stocks, virus recovered from BSR-T7 cells was inoculated on a monolayer of RAW264.7 cells (m.o.i. 0.001) to obtain MNV-3 p1 (passage 1). Serial passage of MNV-3 (passages 2–6) was carried out in RAW264.7 cells (m.o.i. 0.1–0.3). Infected cells were then incubated at 37 °C until cytopathic effect was apparent (typically 48 h) and then subjected to two consecutive cycles of freezing and thawing and subsequent filtering to obtain infectious particles. Virus titres were determined by TCID_50_ assays in RAW264.7 cells for 5 days followed by visual inspection.

#### Establishment of persistent MNV infections *in vivo*.

Groups of six C57BL/6 male mice of 4–5 weeks of age (Harlan or Charles River) were inoculated by oral gavage with 100 µl sample containing varying amounts of MNV-3 virus stocks. Mock-infected animals were administered either filtered cell-culture lysates or 10^5^ TCID_50_ of MNV-3 previously inactivated by UV exposure. For UV inactivation, 1.6 ml containing 2×10^7^ TCID_50_ of MNV-3 was cross-linked under high-intensity UV light at 4 °C using a Spectrolinker XL-1500 (Spectronics Corporation).

#### TCID_50_ titration of viral samples obtained from animal faeces.

Stool pellets collected from infected animals were placed on ice and dispersed into PBS to reach a final concentration of 50 mg ml^−1^. Resuspended faeces were then centrifuged at maximum speed (15 000 ***g***) for 5 min and 100 µl supernatant was subjected to a second centrifugation step to remove any traces of faecal debris. The double-purified samples were titrated by TCID_50_ assay in RAW264.7 cells.

#### RNA extraction and RT-PCR-based detection of MNV-3 in faeces and tissues.

Viral RNA was extracted from 100 µl supernatant of faeces dispersed into PBS (50 mg ml^−1^) and from an approximately 20 mg portion of each tissue following the indications provided with the GenElute Mammalian Total RNA Miniprep kit (Sigma-Aldrich). Different tissues/organs collected were stored in RNAlater solution (Ambion) at −80 °C until RNA extraction was performed. For semiquantitative detection of MNV-3 RNA, one- or two-step RT-PCRs were performed using primers spanning genomic residues 3395–3430 (forward) and 3770–3734 (reverse) (reference GenBank accession no. JQ658375), and Moloney murine leukemia virus (M-MLV) reverse transcriptase and Go*Taq* DNA polymerase (Promega). The resulting 376 bp product was then resolved on a 2 % agarose gel. To obtain an accurate determination of the number of MNV-3 RNA molecules in faecal or tissue samples, we carried out reverse transcription–quantitative PCR following protocols described previously for the broad detection of MNV strains (Kitajima *et al.*, 2010). Reverse transcription of MNV cDNA was performed using M-MLV reverse transcriptase (Promega) and a primer complementary to genomic positions 5345–5380. MNV cDNA was then quantified by quantitative PCR with primers spanning residues 5028–5047 (sense) and 5177–5138 (antisense), and a TaqMan FAM-TAMRA-labelled probe complementary to residues 5077–5062. Quantitative PCR determinations were carried out with Precision 2× qPCR MasterMix (Primerdesign) in a ViiA7 Real-Time PCR system apparatus (Applied Biosystems). In all the experiments, a standard curve for MNV RNA with a known number of molecules was carried out in parallel.
